# Young children can use their subjective straight-ahead to remap visuo-motor alterations

**DOI:** 10.1038/s41598-023-33127-w

**Published:** 2023-04-20

**Authors:** Davide Esposito, Jenifer Miehlbradt, Alessia Tonelli, Alberto Mazzoni, Monica Gori

**Affiliations:** 1 Unit for Visually Impaired People, Fondazione Istituto Italiano di Tecnologia, 16163 Genova, Italy; 2 Bertarelli Foundation Chair in Translational Neuroengineering, EPFL, 1015 Lausanne, Switzerland; 3 The Biorobotics Institute, Scuola Superiore Sant’Anna, 56127 Pontedera, Italy

**Keywords:** Sensorimotor processing, Navigation, Biomedical engineering, Human behaviour

## Abstract

Young children and adults process spatial information differently: the former use their bodies as primary reference, while adults seem capable of using abstract frames. The transition is estimated to occur between the 6th and the 12th year of age. The mechanisms underlying spatial encoding in children and adults are unclear, as well as those underlying the transition. Here, we investigated the role of the subjective straight-ahead (SSA), the body antero-posterior half-plane mental model, in spatial encoding before and after the expected transition. We tested 6–7-year-old and 10–11-year-old children, and adults on a spatial alignment task in virtual reality, searching for differences in performance when targets were placed frontally or sideways. The performance differences were assessed both in a naturalistic baseline condition and in a test condition that discouraged using body-centered coordinates through a head-related visuo-motor conflict. We found no differences in the baseline condition, while all groups showed differences between central and lateral targets (SSA effect) in the visuo-motor conflict condition, and 6–7-year-old children showed the largest effect. These results confirm the expected transition timing; moreover, they suggest that children can abstract from the body using their SSA and that the transition underlies the maturation of a world-centered reference frame.

## Introduction

Spatial information processing is a complex ability that must deal with the human behavior's dynamic nature. Evidence shows the brain may rely on an abstract construct, external to any body part, to encode the object's egocentric location, i.e., the object's location relative to the observer^1–3^. For example, Waller et al.^3^ found that the smallest pointing errors in a spatial memory task lie along the line between object and observer regardless of the head or trunk position, suggesting that this line (or a similar construct) acted as an egocentric reference frame. The external construct most likely to serve as an egocentric reference frame for azimuthal localization (i.e., localization in the horizontal plane) is the subjective straight-ahead (SSA)^4,5^, i.e., the internal representation of the whole body’s antero-posterior sagittal half-plane. Experimental evidence suggests the SSA is a multimodal construct: it can be influenced by vision^6,7^; vestibular information^2,8,9^; proprioception^10,11^; experience and expectation^7,12,13^. It seems acquired through development; indeed, infants start to develop an awareness of body symmetry by 6 months^14,15^. Therefore, since the body's plane of symmetry is *de-facto* the sagittal plane, it is plausible that developing symmetry awareness requires children to know where their plane of symmetry is. Moreover, blind infants show delays in developing the sense of body symmetry^16^, supporting the developmental nature of the SSA and suggesting that vision might play a significant role in such a process.

Notwithstanding the existence of an egocentric mental construct detached from any specific body part, such as the SSA, since early childhood, the ability to use it to program motor actions seems a trait of adulthood. For example, Roncesvalles et al.^17^ tested the posture control of children from 2 to 9 years of age and adults with various trunk-pitch experimental manipulations. Their data showed that the adult-like use of an external reference frame was present only in the 7–9-year-old group, while younger children used their trunk as a reference frame. Whereas Roncesvalles’ study focused on the pitch axis only, Miehlbradt et al. found a similar age trend in a virtual reality-based visuo-motor task involving yaw and roll axes^18^. To create an intuitive method to pilot a drone in a first-person perspective with whole-body movements, Miehlbradt et al. designed a visuo-motor association where a combination of yaw and roll rotations of the head and trunk allowed to steer a virtual drone and the pilot’s field of view (FoV)^19^. Such a steering method was intuitive for adults, but not children aged less than 9, who struggled to orient in the virtual space with the altered visuo-motor association^18^. This result agrees with Roncesvalles’ finding that children do not use an abstract reference frame for egocentric information encoding^17^. However, Miehlbradt et al. employed a biaxial visuo-motor manipulation and did not restrain the FoV movement; hence, the effect may have emerged due to the task complexity rather than the inability to use an abstract reference frame; in addition, the study did not focus on the azimuth encoding. To shed light on the origin of the effect Miehlbradt et al. found, we conducted another study, assessing the ability to cope with an alteration of the head-related visuo-motor association for azimuthal orientation encoding^20^. We assessed the performance of a group of 6-year-old children, 10–11-year-old children, and a group of adults on a first-person perspective steering game where participants drove an arrow towards a target using head or trunk yaw rotations. They repeated the task twice: once with the FoV being controlled by the head, as in daily life (baseline condition), and once with the FoV control shared between the head and trunk, thus penalizing the use of a body-centered reference frame (test condition). In the test condition, 6–7-year-old children performed worse than 10–11-year-old children and adults^20^, in accordance with Roncesvalles and Miehlbradt's results^17,18^.

The studies mentioned above agree that young children struggle with using an unspecific egocentric mental construct, like the SSA, but they did not test directly for the SSA use. In the present study, we evaluated this aspect explicitly by repeating the task used in Esposito et al.'s work^20^, with the same baseline and test conditions, but this time we distinguished between trials where the target appeared in front of the participant's seat and those where the target appeared on the side of the participant's seat. This way, we could assess the SSA involvement in the egocentric azimuth encoding. We hypothesized that if 6–7-year-old children used the trunk as an egocentric reference frame, they should not show differences in the baseline condition’s spatial performance between frontal and lateral targets, whereas 10–11-year-old children and adults, who have previously demonstrated the ability to use an external frame, would. In addition, if the SSA is involved in defining the external egocentric reference frame, the test condition should amplify the effect by making the body-centered frames unreliable.

## Methods and analysis

### Participants

29 children between 6 and 11 years old and 14 adults were tested. Children were recruited from contacts within schools in Genova. Adults were recruited through convenient sampling and a mailing list obtained from the Istituto Italiano di Tecnologia. The study followed the tenets of the Declaration of Helsinki and was approved by the ethics committee of the local health service (Comitato Etico, ASL 3, Genova). Informed consent was obtained from all participants and/or their legal guardian(s). Children were divided into two groups according to their class in school: 6–7-year-old (8 males, 5 females, age = 6.08 ± 0.08 years) and 10–11-year-old (11 males, 5 females, age = 10.10 ± 0.08 years). The average age of the adults (7 men and 7 women) was 33.29 ± 2.88 years.

### Apparatus

The apparatus was the same as in Esposito et al.^20^. The VR platform running the experiment, called VRCR, was developed with the game engine Unity 3D. An Oculus Rift® head-mounted display (HMD) delivered the visual information and tracked the participants' head rotations. Trunk rotations were tracked by a tri axial Xsens® wireless inertial measurement unit (IMU) fixed on their back using a custom-made harness. Data from the IMU were asynchronously collected and stored in a parallel thread via UDP socket and then imported into the main thread at a sampling rate (f_s_) of 50 Hz. Such value is appropriate to track trunk rotations as it is larger than twice the frequency band of human body movements, which is approximately 20 Hz^21^. The head was tracked at a f_s_ of 90 Hz. Such value was due to Unity itself, which forces its main loop to match the refresh rate of the HMD in use. In fact, the refresh rate (f_r_) of the Oculus HMD and Unity's main loop were again 90 Hz. The f_r_ of Unity's physics engine was the standard value of 30 Hz to keep a good tradeoff between performance and computational cost^22^. Rotations were updated at the main loop’s f_r_ to update the visuo-motor associations as fast as possible, while translations were updated at the physics engine’s f_r_ to guarantee constant flying speed^23^. During the experiment, participants were seated on a chair, and they were requested to sit toward the chair’s front and not to lean against the backrest, to let their upper body, head included, free to move.

### Task and stimuli

The stimuli were the same as in Esposito et al.^20^. The VRCR virtual environment is a virtual archery field. The unit of length in Unity-based applications is the Unity Unit (UU). However, the VRCR development kept a 1:1 ratio between UU and meters; hence in the following, the meter is used as a measurement unit for virtual lengths. The camera view was raised from the ground of 1.7 m. The target lay at 60 m and could appear in three positions concerning the participant's seat: − 30°, 0°, + 30°. Only one target was present in each trial to guarantee a purely egocentric task. The arrow was automatically shot when the participant, rotating their body segment as requested by the experimental condition, placed the arrow in a trigger area for a random time between 1 and 3 s. The trigger area was defined as a range of ± 3° around the starting position. It was rendered graphically as a black column. It could appear in the same positions as the target (at − 30°, 0°, or + 30° from the participant's seat), yet it was always at 30° from the active target to avoid the participant could have the arrow hit the target without moving.

Once shot, the arrow traveled with a fixed speed of 15 m/s, and it only moved in the horizontal plane.

**Figure 1:**
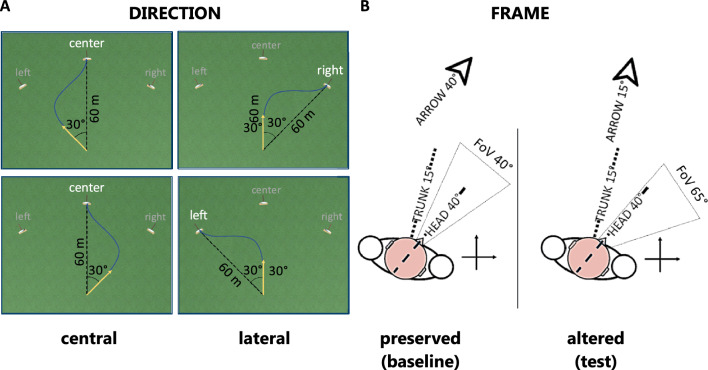
In panel (**A**), elements in the virtual environment and their metric relationships depending on the "direction" factor levels, with examples of possible arrow trajectories (blue full lines). The yellow arrow highlights the starting arrow direction. Targets with dark grey names are inactive during the trial; the only active target is the one with white text. Examples on the same rows show conditions where the relative position of arrow and target is the same, but the absolute target position (i.e., the "direction" level) is different. In panel (**B**), example of relationship between head, trunk, field of view and arrow for the two conditions under investigation in the present experiment.

The study used the same four conditions as in Esposito et al.'s^20^, defined by three factors: “direction”, “control” and “contingency”. The first factor, "direction", rules the relative initial target position concerning the participant's straight-ahead. Its two levels are *central* and *lateral*. When the “direction” is *central*, the target appears in line with the participant's straight-ahead, and the trigger area is at 30 ± 3°, randomly alternated between rightwards and leftwards; when it is *lateral*, the target appears at 30° from the participant's straight-ahead, randomly alternated between rightwards and leftwards, and the trigger area is at 0 ± 3° (Fig. 1A). The second factor, "control", indicates which body segment controls the arrow: the *head* or the *trunk*. When the “control” is *head*, the arrow yaw is the head yaw; when it is the *trunk*, the arrow yaw is the trunk yaw. The third factor, "contingency", rules the causal relationship between body movement and FoV shift, and it has two levels: *normal* and *shared*. When the “contingency” is *normal*, the FoV depends on the head movement, as in everyday life; when it is *shared*, it depends on the head movements plus the trunk-in-space yaw.

The study combined the factors “control” and “contingency” to design a baseline condition that preserves the ecological visuo-spatial reasoning and a test condition that makes the trunk an unreliable reference frame. The baseline condition results from combining *head* “control” and *normal* “contingency”; it implements a simple alignment task by head-pointing with unrestrained trunk. The test condition results from combining *trunk* “control” and *shared* “contingency”; it implements an alignment task by trunk-pointing with altered head-related visuo-motor association. The head-related visuo-motor association alteration occurs because the FoV control is shared between head and trunk and at the same time the participant must move the trunk to drive the arrow. In this way, if the participant tries to drive the arrow towards the target, they will also rotate their FoV. To simplify the VRCR conditions naming, the following text will treat the baseline and the test conditions as two levels of the factor "frame": the baseline condition will be the *preserved* level; the test condition will be the *altered* level (Fig. 1B). The study compared the performance between *central* and *lateral* “direction” levels in the *preserved* and *altered* frame conditions.

It is worth noting that the conditions used in this study differed only in how the participant’s movements were mapped onto the virtual environment, and no physical restraints were used.

### Experimental procedure

Before starting the experiment, the researchers ensured the participants knew what archery is. Then, the participants were introduced to the experiment with the following sentence: "you will play an archery-like game. But, unlike the real archery, you will not simply shoot the arrow: you will be on it. And you will control it by turning your head, your trunk, or a combination of them". Then, the participants performed a short training for the *preserved* and *altered* "frame" levels to familiarize themselves with the task and to ensure they could perform it. The "direction" level was chosen randomly. The trials for the training session did not exceed 6 trials per training condition. The video recording of one training session is provided as supplementary material S1 to show the experimental setting and clarify how the training sessions were conducted. The legal guardian of the participant in the video gave informed consent for publication of identifying information/images/videos from the experimental session for scientific, informative, and/or institutional purposes.

Each experiment consisted of 4 blocks corresponding to the two "frame" levels repeated in the two "direction" levels. To prevent the learning effect, a randomized partial counterbalanced design was used. Each block comprised 8 trials. Before proceeding with a block, the experimenter told the participant what condition they had been doing and explained the movement and the direction the following block’s condition demanded. If the participant struggled with finding the starting position, the experimenter helped them via verbal guidance. No help was given in any case to the participant after the arrow was shot. At any time, the participant could take a break. For the 6–7-year-old children, even though their tolerance for commercial VR headsets is adult-like for sessions as long as 30 min^24^, break time was made every 15 min to avoid straining their neck muscles, and every other time they asked for one. The whole data collection for one participant lasted between 30 and 45 min, including breaks.

### Statistical analysis

**Figure 2:**
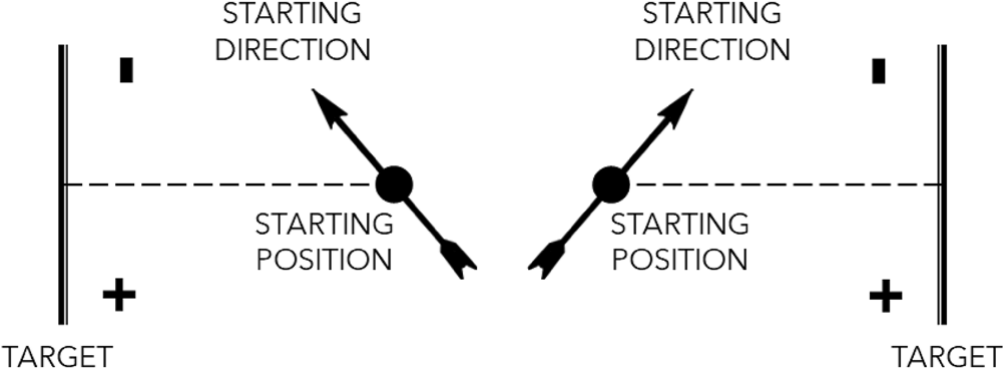
Schematic representation of the logic behind the final arrow position sign assignment. The sign was negative if the arrow ended its run in the same hemi-field from which it started, in other words if it undershot the target; it was positive otherwise. The scheme shows two arrow-target configurations. In absolute coordinates, the final arrow position signs should be positive above the target center in one configuration and positive below in the other. Instead, since the arrows start oriented towards the upper hemi-field in both the configurations, the positive signs lie below the target center in both the configurations.

The study compared spatial constant and variable errors in hitting the target center between central and lateral targets in each "frame" level. Such comparison aimed to evaluate the SSA involvement in egocentric azimuth encoding. Constant and variable errors were extracted from the error distribution, i.e., the distribution of distances from the target center. The error signs were corrected to have negative values when the participant undershot the target eccentricity with respect to their starting direction or positive if they overshot, as shown in Fig. 2. The constant error was computed as the median of the error distribution; its interquartile range (IQR) estimated the variable error. Nonparametric estimates were chosen because they reduce the effect of extreme samples compared to Gaussianity-based estimates such as mean and variance, and this is desirable when the sample size is small, as it is in our case (8 trials per condition). The study used a threshold on the constant error as exclusion criterion. The threshold was set to − 27 m, that is, the error obtained if the arrow were shot at 27° (27 = 30–3, see subsection "Task and stimuli") from the target and never steered: crossing such value was treated as indicator of the participant not performing the task. Reaching the threshold in any condition determined the exclusion of the participant’s whole data. The minimum constant error in our dataset was − 18 m, therefore no participant was excluded.

**Figure 3:**
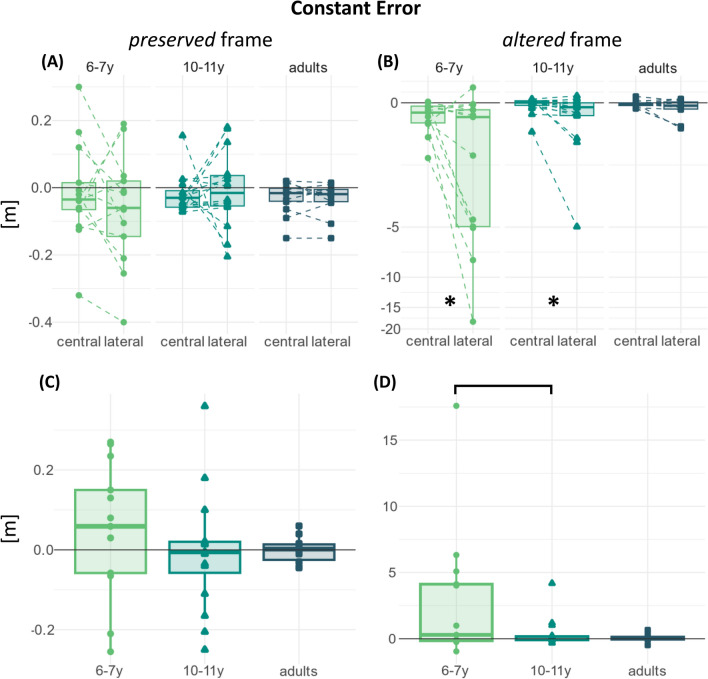
Raw (**A**,**B**) and differential (**C**,**D**) constant error distributions, split according to the “frame” factor levels: preserved (**A**,**C**) and altered (**B**,**D**). The y-axes in panels (**A**) and (**B**) are in the 10^0^ magnitude scale. The y-axes in panels (**C**) and (**D**) are in the 10^1^ magnitude scale, and they have been transformed in pseudo-logarithmic scale for the sake of data visualization clarity. The asterisks indicate the significant within-age comparisons. The bars indicate the significant between-age comparisons.

**Figure 4:**
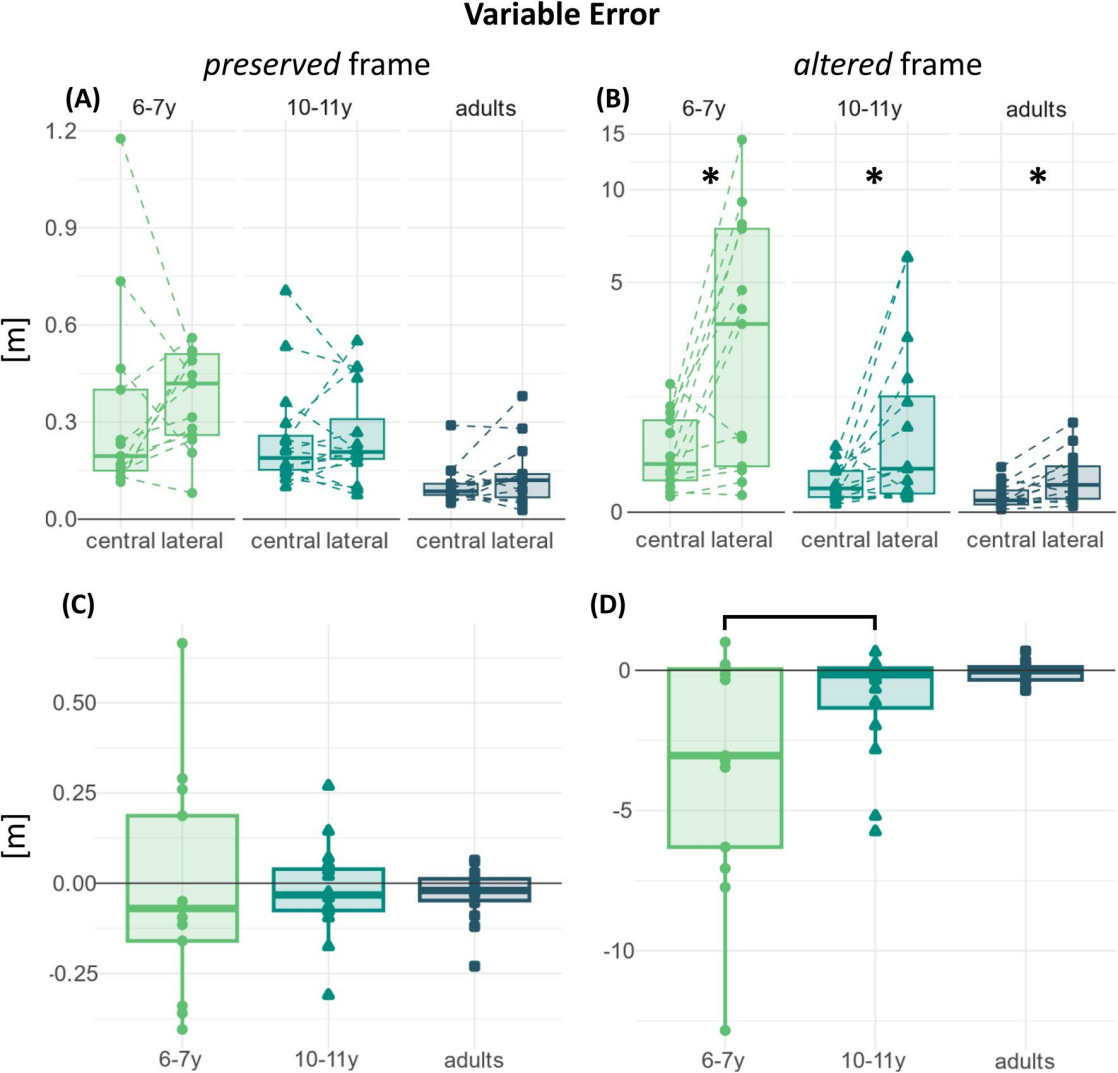
Raw (**A**,**B**) and differential (**C**,**D**) variable error distributions, split according to the “frame” factor levels: preserved (**A**,**C**) and altered (**B**,**D**). The y-axes in panels (**A**) and (**B**) are in the 10^0^ magnitude scale. The y-axes in panels (**C**) and (**D**) are in the 10^1^ magnitude scale, and they have been transformed in pseudo-logarithmic scale for the sake of data visualization clarity. The asterisks indicate the significant within-age comparisons. The bars indicate the significant between-age comparisons.

The samples' Gaussianity was assessed via Shapiro–Wilk tests. The tests rejected the null hypothesis of Gaussianity; therefore, non-parametric statistics were used. Rank-based statistics were not appropriate, because of the high number of ties in the ranking procedure. Therefore, the study used permutation-based tests. The study tested for the presence of an SSA-mediated effect by means of a set of mixed ANOVAs. A first omnibus ANOVA tested all factors ("group”,”direction”,”frame”); then, if the three-way interaction factor was significant, two follow-up ANOVAs tested the factors “group” and “direction” on the two data subsets obtained by splitting the dataset by the levels of the “frame” factor. The post-hoc comparisons for the within-age effect (*central* vs *lateral* as main effect or split by age-group) used paired t-tests, coupled with rank-biserial correlation to estimate the standardized effect size^25^. The study did not perform any post-hoc test on the between-age effects, either overall or within each “direction” level, because such comparisons do not probe age-related changes in the SSA-mediated effect, but rather changes in the (overall or “direction”-specific) task performance, which do not fit in this study’s aim. Furthermore, the overall developmental change was reported in a previous study^20^. Instead, the study analyzed the within-participant performance differences between *central* and *lateral* directions in each “frame” level to assess the developmental change in the SSA-mediated effect. The choice of analyzing the differences between *central* and *lateral* “direction” levels aimed to probe the SSA-mediated facilitation effect directly, suppressing at the same time the effect of any age-related confounding factors that may affect the performance in the *central* and *lateral* “direction” equally, such as the ergonomic (dis)comfort in using the VR headset. The Gaussianity of this new sample set was assessed via Shapiro–Wilk tests. The tests rejected the null hypothesis of Gaussianity. Moreover, the visual inspection of data distributions (Figs. 3 and 4) showed high heteroscedasticity. To cope with non-Gaussianity and heteroscedasticity, the study used permutation-based weighted linear regression^26^, with the "group" factor as a predictor. The “group” levels were encoded using dummy (or treatment) contrast coding^27^ and "10–11-year-old" children as reference level to test for the presence of developmental changes in the SSA-mediated effects before and after that age. The model coefficients' significances were assessed via permutation tests. The study performed the analyses on the developmental change in the SSA-mediated effect as post-hoc tests of the follow-up ANOVAs, providing that the latter tests reached significance for the “group:direction” interaction effect.

Data cleaning was performed in MATLAB R2015b^28^; data visualization and analysis were performed in R^29^. Shapiro–Wilk tests were done with the built-in R function "shapiro.test"^29^. Rank-biserial correlations were computed via the function "rank_biserial" from the R package “effectsize”^30^. Permutation-based ANOVAs were performed via the function “aovperm” from the package “permuco”^31^. Permutation-based weighted linear regressions were performed via the function “lmp” from the package “lmPerm”^32^. The plots reported in the following chapter were generated via the R package “ggplot2”^33^.

## Results

The study aimed to assess the developmental change in the SSA involvement to encode egocentrically azimuthal orientation. To do so, it compared constant error and variable error in hitting the target when the latter was placed in front of the participant or sideways*,* for different age groups, with reliable or unreliable embodied egocentric frame. The following section will present, for each metric under investigation, the results of the omnibus ANOVA, eventually followed by the results of the follow-up ANOVAs, eventually followed by the post-hoc tests, first the within-age ones comparing the *central* and *lateral* “direction” levels per se, then the between-age ones comparing the differences between *central* and *lateral* “direction” levels. Raw data distributions, paired according to the “direction” factor, are graphically reported in Fig. 3. Differential data distributions are graphically reported in Fig. 4.

### Constant error

Constant error values correspond to the median of the distances between arrow endpoint and target center. Negative values mean the arrow undershot the target with respect to the starting point and vice-versa.

The omnibus mixed ANOVA test was significant for all the effects tested. The results are reported in Table 1. The following sub-sections present the results for each “frame” factor’s level.

**Table 1:** Results of the permutation-based mixed ANOVAs performed on the constant error distributions.

	SSn	dfn	SSd	dfd	MSEn	MSEd	F	parametric P(> F)	resampled P(> F)	η_p_^2^
Constant error										
Omnibus										
Group	25.61	2	101.14	40	12.81	2.53	5.07	0.011	0.001	0.202
Direction	14.68	1	81.12	40	14.68	2.03	7.24	0.010	0.002	0.153
Frame	21.89	1	91.27	40	21.89	2.28	9.59	0.004	0.000	0.193
Group:direction	16.04	2	81.12	40	8.02	2.03	3.96	0.027	0.008	0.165
Group:frame	24.63	2	91.27	40	12.31	2.28	5.40	0.008	0.001	0.213
Direction:frame	14.01	1	78.91	40	14.01	1.97	7.10	0.011	0.002	0.151
Group:direction:frame	14.76	2	78.91	40	7.38	1.97	3.74	0.032	0.011	0.158
“frame” *preserved*										
Group	0.01	2	0.55	40	0.01	0.01	0.37	0.691	0.704	0.018
Direction	0.00	1	0.33	40	0.00	0.01	0.47	0.498	0.511	0.012
Group:direction	0.02	2	0.33	40	0.01	0.01	0.93	0.404	0.402	0.044
“frame” *altered*										
Group	50.23	2	191.90	40	25.11	4.80	5.24	0.010	0.001	0.208
Direction	28.68	1	159.70	40	28.68	3.99	7.18	0.011	0.001	0.152
Group:direction	30.78	2	159.70	40	15.39	3.99	3.86	0.029	0.009	0.162

Parametric p-values are those computed using the classic analytical estimation, while resampled p-values are those computed via permutations. Since the data do not respect the assumptions for parametric ANOVAs, this study considered the permutation-based p-values.

#### Preserved “frame”

The follow-up mixed ANOVA did not reach significance in any of the effects tested. The results are reported in Table 1.

#### Altered “frame”

The follow-up mixed ANOVA reached significance in all the effects tested. The results are reported in Table 1.

The permutation-based paired t-test reached significance in the 6–7-year-old group, *t(12)*_6–7_ = 2.08, *p* = 0.026, *r*_rb_ = 0.71, 95% CI [0.27, 0.91], and in the 10–11-year-old group, *t(15)*_10–11_ = 1.70, *p* = 0.048, *r*_rb_ = − 0.50, 95% CI [− 0.04, 0.81], while in the adult group it approached significance, *t(13)*_adults_ = 2.04, *p* = 0.060, *r*_rb_ = 0.50, 95% CI [− 0.04, 0.81].The permutation test on the coefficients of the weighted linear model fitted on the differences between *central* and *lateral* “direction” levels reached significance in the “6–7-year-old vs 10–11-year-old” coefficient,* c*_*6-7*|*10–11*_ = 2.46, *p* = 0.005, but not in the “10–11-year-old vs adults” coefficient, *c*_*10-11*|*adults*_ = − 0.28, *p* = 0.350.

Overall, the test results indicated that the alteration of the head-related visuo-motor association for heading exposed a significant SSA-mediated effect on the 6–7-year-old group’s constant error, which was significantly larger than in the 10–11-year-old group. The 10–11-year-old group exposed a significant SSA-mediated effect on their constant error, but it was not significantly different from the adults’ one.

### Variable error

Variable error values correspond to the IQR of the distances between arrow endpoint and target center. Small values mean the arrow endpoints were close to each other, and vice-versa.

The omnibus mixed ANOVA test was significant for all the effects tested. The results are reported in Table 2. The following sub-sections present the results for each “frame” factor’s level.

**Table 2:** Results of the permutation-based mixed ANOVAs performed on the variable error distributions.

	SSn	dfn	SSd	dfd	MSEn	MSEd	F	parametric P(> F)	resampled P(> F)	η_p_^2^
Variable error										
Omnibus										
Group	42.73	2	86.61	40	21.36	2.17	9.87	0.000	0.000	0.330
Direction	27.21	1	67.37	40	27.21	1.68	16.15	0.000	0.000	0.288
Frame	48.51	1	77.99	40	48.51	1.95	24.88	0.000	0.000	0.383
Group:direction	17.20	2	67.37	40	8.60	1.68	5.11	0.011	0.005	0.203
Group:frame	29.19	2	77.99	40	14.59	1.95	7.48	0.002	0.002	0.272
Direction:frame	25.49	1	61.94	40	25.49	1.55	16.46	0.000	0.000	0.292
Group:direction:frame	16.78	2	61.94	40	8.39	1.55	5.42	0.008	0.004	0.213
“frame” *preserved*										
Group	0.73	2	1.60	40	0.37	0.04	9.14	0.001	0.001	0.314
Direction	0.01	1	0.71	40	0.01	0.02	0.79	0.380	0.363	0.019
Group:direction	0.00	2	0.71	40	0.00	0.02	0.13	0.881	0.884	0.006
“frame” *altered*										
Group	71.18	2	163.00	40	35.59	4.08	8.73	0.001	0.000	0.304
Direction	52.69	1	128.60	40	52.69	3.22	16.39	0.000	0.000	0.291
Group:direction	33.97	2	128.60	40	16.99	3.22	5.28	0.009	0.006	0.209

Parametric p-values are those computed using the classic analytical estimation, while resampled p-values are those computed via permutations. Since the data do not respect the assumptions for parametric ANOVAs, this study considered the permutation-based p-values.

#### Preserved “frame”

The follow-up mixed ANOVA reached significance in the “group” main effect only, *F(2,40)* = 9.14, *p* < 0.001, ${\upeta }_{p}^{2}$=0.31. The results are reported in Table 2.

#### Altered “frame”

The follow-up mixed ANOVA reached significance in all the effects tested. The results are reported in Table 2.

The permutation-based paired t-test reached significance in every age group, *t(12)*_6–7_ = − 2.94, *p* = 0.016, *r*_rb_ = − 0.84, 95% CI [− 0.95, − 0.55]; *t(15)*_10–11_ = − 2.35, *p* = 0.028, *r*_rb_ = − 0.76, 95% CI [− 0.91, − 0.41]; *t(13)*_adults_ = − 3.33, *p* = 0.007, *r*_rb_ = − 0.81, 95% CI [− 0.94, − 0.49].

The permutation test on the coefficients of the weighted linear model fitted on the differences between *central* and *lateral* “direction” levels reached significance in the “6–7-year-old vs 10–11-year-old” coefficient,* c*_*6-7*|*10–11*_ = − 2.23, *p* = 0.005, but not in the “10–11-year-old vs adults” coefficient, *c*_*10-11*|*adults*_ = 0.97, *p* = 0.250.

Overall, the test results indicated that the alteration of the head-related visuo-motor association for heading exposed a significant SSA-mediated effect on the variable error of all the age groups. Such effect was significantly larger in the 6–7-year-old group than in the 10–11-year-old group. The 10–11-year-old group’s effect was not significantly different from the adult group’s one.

## Discussion

The present study investigated the developmental time-course of using the mental representation of the straight-ahead direction, called subjective straight-ahead (SSA), to encode egocentric azimuthal orientation. To do so, differences in spatial constant and variable error on a visuo-spatial alignment task were compared between trials with a frontal target and trials with a lateral target in a 6–7-year-old children, 10–11-year-old children, and adults. The baseline condition implemented a naturalistic head-related visuo-motor association, designed to leave the egocentric encoding untouched. The test condition induced a visuo-motor alteration of the head-related visuo-motor association, designed to penalize the use of the trunk as a reference frame. No SSA effect was detected in the baseline condition in any group; contrarily, all age groups showed the SSA effect on their variable error in the test condition. Moreover, 6–7-year-old children showed a significantly larger SSA-mediated effect than 10–11-year-old children concerning both constant and variable errors.

Overall, these results show a significant behavioral change between the ages of 6–7 and 10–11, in agreement with the literature that identifies a transition in egocentric processing in the same age span^17,18^. At the same time, they reject our hypothesis that children younger than 10–11 do not rely on the SSA to encode azimuthal orientation. Indeed, we interpret our results as evidence that the 6–7-year-old children already have their SSA representation, and they anchor to it when their embodied egocentric reference frame becomes unreliable, as in the test condition of this study. Instead, 10–11-year-old children and adults showed smaller but consistent performance differences between *central* and *lateral* directions. The smaller SSA-mediated effect in the two older populations indicates an age-related improvement in compensating for the visuo-motor alteration corrupting the trunk’s role as egocentric frame. This improvement may reflect an enhancement in the ability to abstract their egocentric frame from their body. However, the presence of the effect in the older populations suggests that such an external reference frame includes the SSA to some degree. The external reference frame under consideration may be an egocentric mental construct, like the SSA or the ideal line between observer and object^3^, or even an allocentric reference frame, a reference frame independent from the observer's point of view^34^. Indeed, several authors identified interaction effects between egocentric and allocentric reference frames when the stimuli were close to the participants' straight-ahead direction^35,36^, suggesting that the SSA may be involved in defining both reference frame types. Fink et al.^37^ investigated the interaction between egocentric and allocentric frames, and found modifications in the cortical activity of participants performing allocentric judgments in the line bisection task while their egocentric space was distorted by galvanic vestibular stimulation. However, the neurofunctional difference did not show up in the behavioral results. A similar compensatory effect may explain the smaller differences in constant and variable errors between frontal and lateral targets in 10–11-year-old children and adults. Therefore our results suggest that the external reference frame exposed in this study is an allocentric reference frame that interacts with the SSA to perform egocentric judgments rather than an egocentric mental construct. In light of this interpretation, the different behavior found in 6–7-year-old children could reflect their struggle with the task of merging egocentric and allocentric cues^38^, or their struggle with allocentric reasoning per se^39–41^. Indeed, it has been shown that the brain can associate information to extract high-level features, such as the spatial cues^42,43^, in different ways^44^, and that the same pieces of information are merged differently at different ages^45^. This difference can arise because of a change in the underlying algorithm used by the brain, that is, a shift from alternation to integration of redundant cues^38,46^, or because of a change in the weighting of (redundant and non-redundant) lower-level information^47,48^. The SSA is a multi-modal construct that arises from merging interoceptive and exteroceptive cues that can be both redundant (e.g., visual, acoustic, and somatic midlines while standing straight-ahead) and non-redundant (e.g., visual, acoustic, and somatic midlines while turning). At the same time, there is evidence that egocentric and allocentric cues can be merged into a final percept in a similar way as it happens for the extraction of multi-modal cues from the uni-modal ones^47^. The present data do not provide any mean to discern at what processing stage the developmental change takes place; however, they provide clear evidence of a strict link between the maturation of spatial abilities and that of multi-modal information processing. One interesting implication of such a link resides in the existence of a phenomenon known as cross-modal calibration^45,48^: in the presence of two redundant cues, the most informative one calibrates the other. Recently, it has been proposed that the ability to integrate the cues, and therefore the ability to generate multi-modal constructs, may be a prerequisite for cross-modal calibration to take place. Translating this concept to our data, we speculate that the SSA may be seen as the multi-modal construct needed to let the cross-modal calibration of spatial cues happen, that is, the SSA may act as a catalyst for the calibration of allocentric reasoning by means of the egocentric cues. Future investigations should delve into the relationship between allocentric abilities, effects related to the interaction between egocentric and allocentric abilities, and SSA-mediated effects, to clarify the causal links that shape such a complex system.

One may argue that the age-related differences may be due to optical mismatches between HMD and children's eyes, HMD instability on the children's heads, or even the younger children failing to understand the task. Even though we cannot exclude these confounding factors with absolute certainty, the odds that they affected our data are extremely low. By contradiction, age-related differences should have been present in the *preserved* condition as well. Moreover, the "direction" factor only manipulates the initial body position: the visual scene displayed is the same in the *central* and *lateral* "direction" levels. Therefore, the effects are likely attributable to differences in the spatial information processing strategy used at different ages rather than differences in visual stimuli or task comprehension.

The present study did not monitor gaze, a known source of spatial distortions in eccentricity estimation^1,49,50^, but assumed that the participants would keep their gaze on the target, the only item in the virtual environment. Since the targets were placed at the same FoV-centered coordinates in the two "direction" levels, the study assumed equally distributed eye movements; however, without eye-tracking data, an association between the effects found and differences in the pattern of eye movements cannot be excluded. Repeating the present paradigm with eye-tracking in future studies should resolve such ambiguity.

Another limitation of this study is it did not consider the participants' experience using VR devices. We expect such an effect to be weak since the requested gestures were ubiquitous (head turns) or never undertaken (commercially available VR games, by design, try to preserve the naturalistic visuo-motor associations to reduce discomfort and sickness^51,52^). Nevertheless, we cannot exclude its presence in our results.

Finally, this study did not use a virtual avatar to prevent the avatar from acting as an allocentric reference frame. Nevertheless, a look-alike virtual avatar inside an immersive VR system has been shown to have essential effects on the sense of presence, as well as on the interaction with, and the perception of, the virtual space^53^. These effects have been found mainly in height^54^ and depth^55^ estimation, while this work probed azimuthal orientation only. However, future work should aim to understand the influence of virtual avatars on participants' performance in the tasks presented here.

In conclusion, the pattern of results found in the present study suggested that, in contrast with the literature about egocentric spatial abilities development, the SSA, an out-of-body egocentric mental construct, can facilitate egocentric encoding already at the age of 6–7, provided that the embodied reference frames become unreliable. Instead, 10–11-year-old children and adults in the same condition used an allocentric reference frame interacting with the SSA to perform an egocentric judgment.

The pervasive and early involvement of the SSA in the egocentric computation to cope with the embodied egocentric frame unreliability suggests that the SSA may be the bridge between egocentric and allocentric reasoning in adulthood and an egocentric catalyst that calibrates allocentric reasoning in childhood. Future studies are necessary to investigate the relationship between SSA and reference frames in the brain and unveil how eye movements contribute to the SSA effect.

## Supplementary Information

Supplementary Video 1.

Supplementary Information 1.

Supplementary Video 2.

## Data Availability

The clean dataset and the R codes for statistical analyses are available in the “Open Science Framework” repository at the link “https://www.doi.org/10.17605/OSF.IO/G7SK3”. Raw and intermediate datasets are available from the corresponding author upon reasonable request.
