# Author Correction: Young children can use their subjective straight-ahead to remap visuo-motor alterations

**DOI:** 10.1038/s41598-023-36805-x

**Published:** 2023-06-21

**Authors:** Davide Esposito, Jenifer Miehlbradt, Alessia Tonelli, Alberto Mazzoni, Monica Gori

**Affiliations:** 1 Unit for Visually Impaired People, Fondazione Istituto Italiano di Tecnologia, 16163 Genova, Italy; 2 Bertarelli Foundation Chair in Translational Neuroengineering, EPFL, 1015 Lausanne, Switzerland; 3 The Biorobotics Institute, Scuola Superiore Sant’Anna, 56127 Pontedera, Italy

Correction to: *Scientific Reports* 10.1038/s41598-023-33127-w, published online 20 April 2023

The original version of this Article contained errors in the names of authors Davide Esposito, Jenifer Miehlbradt, Alessia Tonelli, Alberto Mazzoni, and Monica Gori, which were incorrectly given as Esposito Davide, Miehlbradt Jenifer, Tonelli Alessia, Mazzoni Alberto and Gori Monica.

The original Article has been corrected.

